# Publicly Funded Home and Community-Based Care for Children With Medical Complexity: Protocol for the Analysis of Medicaid Waiver Applications

**DOI:** 10.2196/13062

**Published:** 2019-07-25

**Authors:** Jessica Keim-Malpass, Lisa C Letzkus, Leeza Constantoulakis

**Affiliations:** 1 University of Virginia School of Nursing Charlottesville, VA United States

**Keywords:** Medicaid, children with medical complexity, home and community-based services, policy analysis, economic evaluation

## Abstract

**Background:**

Children with medical complexity are a group of children with multiple chronic conditions and functional limitations that represent the highest health care utilization and often require a substantial number of home and community-based services (HCBS). In many states, HCBS are offered to target populations through 1915(c) Medicaid waivers. To date, no standard methods or approaches have been established to evaluate or compare 1915(c) waivers across states in the United States for children.

**Objective:**

The purpose of this analysis was to develop a systematic and reproducible approach to evaluate 1915(c) Medicaid waivers for overall coverage of children with medical complexity.

**Methods:**

Data elements were extracted from Medicaid 1915(c) approved waiver applications for all included waivers targeting any pediatric age range through October 31, 2018. Normalization criteria were established, and an aggregate overall coverage score was calculated for each waiver.

**Results:**

Data extraction occurred in two phases: (1) waivers that were considered nonexpired through December 31, 2017, and (2) the final sample that included nonexpired waivers through October 31, 2018. A total of 142 waivers across 45 states in the United States were included in this analysis. We found that the existing adult HCBS taxonomy may not always be applicable for child and family-based service provision. Although there was uniformity in the Medicaid applications, there was high heterogeneity in how waiver eligibility, transition plans, and wait lists were defined. Study analysis was completed in January 2019, and after analyzing each individual waiver, results were aggregated at the level of the state and for each diagnostic subgroup. The published results are forthcoming.

**Conclusions:**

To our knowledge, this is the first study to systematically evaluate 1915(c) Medicaid waivers targeting children with medical complexity that can be replicated without the threat of missing data.

**International Registered Report Identifier (IRRID):**

RR1-10.2196/13062

## Introduction

Children with medical complexity (CMC) are a growing population of medically fragile children (between birth and the age of 21 years) with complex, multisystem disease states; technology dependence; severe functional limitations; complicated treatment regimens and therapies; high utilization of care; and numerous surgical interventions [1-5]. CMC are believed to be extremely susceptible to inequities in health due to access limitations and extreme out-of-pocket financial burden for families [6]. Caring for CMC within a fragmented health care system can be challenging for health care providers [7,8]. Because of numerous hospitalizations, CMC must have care transitions that are coordinated from intensive and acute care settings to ambulatory and community health resources and home care [9]. Caring for CMC at home is a resource- and emotionally intensive experience for families and often results in one partner remaining at the home to provide 24-hour care [3,5,10-13].

There are long-term care funding opportunities for home- and community-based care of CMC; however, each state interprets the eligibility and service provision differently. In many states, long-term care services and support for CMC are provided through the Medicaid Home and Community-Based Services (HCBS) 1915(c) Waivers (implemented through section 1915 of the Social Security Act) and are named such because they allow states to waive certain Medicaid eligibility criteria [14]. HCBS waivers provide states the flexibility to define populations that are high risk based on age and medical condition(s) and to disregard income and resource rules that are traditionally used for Medicaid qualification [15]. All waiver programs must not cost the federal government any more than that if the states did not have the waiver (ie, cost neutrality) [16]. In order to guarantee cost neutrality, states often limit the number of people served under a waiver [16]. Based on the 2013 data, all states reported using cost control measures when implementing the 1915 waivers, such as restrictive functional limitation standards, enrollment limits, or waiting lists, and the average waiting time for services exceeded 2 years [17]. Complicating this financing structure is the fact that children requiring HCBS can be covered through different sources of public and private insurance, which makes overall coverage determination challenging to assess from a policy context [18-25].

To date, no systematic evaluations exist for the Medicaid waiver programs targeted toward children, and there is limited guidance for state policy development and implementation. Previous economic and policy evaluations of the HCBS waiver programs have primarily focused on adult populations and even then, the literature has been incredibly sparse [26,27]. To our knowledge, there is only one systematic evaluation that included services targeted to children and specifically focused on evaluation of 1915(c) waivers for children who received a diagnosis of autism [15,28,29]. Proof-of-concept economic and policy evaluations exist for individual components of home and community-based Medicaid waivers for adults, but there are virtually no data on how various states interpret coverage of services for CMC [14,26]. Given the paucity of data evaluating state Medicaid waivers for children with the most intensive medical needs, this study will facilitate a formal policy evaluation and analysis supporting a comparative approach to evaluate scope of services. Therefore, the purpose of this analysis was to develop a systematic and reproducible approach to evaluate the scope of coverage and services offered through 1915(c) Medicaid waivers for children.

## Methods

### Study Design

This study used a cross-sectional comparative analysis approach involving secondary data collected from 1915(c) Approved Applications that are stored on the Medicaid state waiver website [30]. Each state’s Medicaid office initiates an application for individual waivers to the Centers for Medicare & Medicaid Services, where each application is over 300 pages long and has a uniform structure. Once they are approved, most are considered active for 5 years.

The 1915(c) waivers were included in this analysis if they included children (ages 0-21 years) in the age eligibility criteria across any of the following subgroups that can be defined under the waiver of Section 1902(a)(10)(B) of the Social Security Act: disabled-general (physical or other); disabled-other subgroup (medically fragile, technology dependent, brain injury, and HIV/AIDS), intellectual disability/developmental disability (autism, intellectual disability, and developmental disability), and mental illness (serious emotional disturbance). Relevant waivers were included in this study if they were current and had not expired by October 31, 2018.

### Data Extraction Process and Variable Transformation

We used a systematic data extraction template to ensure uniformity in the process followed by the three authors (Textbox 1). Elements that were included in the abstraction and analysis included pediatric age ranges, ability to transition to adult care services, cost neutrality components (individual cost limits and capitation), individual services offered through the waiver, ability of time-eligible clients to stay on the waiver, and dollars allocated per person. Scope of services were specifically defined using standard HCBS taxonomy including case management; education services; environment, home, or vehicle modifications; specialized equipment; counseling support for the child; counseling support for the caregiver; personal care/day habilitation; respite care; therapies; and skilled or private duty nursing. These domains were chosen based on theory-based clinical relevance and elements central to the administration and policy relevance of the waivers themselves (ie, overall economic allocation of dollars per individual for an amount of time). Due to the heterogeneity in how states define enrollment and transition plans, the data were maintained as the original text data for subsequent secondary qualitative content analysis [31].

Criteria that were obtained for normalization and thus could be compared across states are defined in Textbox 1 along with the source location in the Medicaid waiver application. All variable transformations are also described in Textbox 1. Two-thirds of the waivers had two reviewers to ensure quality control in the data extraction process, with 100% concordance. One advantage of our methodological approach is that the normalization criteria and coverage score calculation can be achieved without the threat of missing data, because all the elements are required components of the waiver applications. Despite this, we are limited in this approach on sole reliance on the elements provided in the waiver applications and the projected spending and enrollment per waiver rather than actual spending and enrollment. Finally, an additional limitation was that wait list information could not be incorporated in the normalization score due to heterogeneity in how states report wait list numbers.

**Final criteria used and variables created for waiver scores that were compared across 1915(c) waivers.**

Domain (original data abstraction in 1915 [c] waiver application) and features and operationalization

**Descriptive feature (Section 1, Request information A-F)**

- State
- Waiver name
- Expiration
- Level of care

**Target group (Appendix B-1: Specification of the Waiver Target Group[s])**

- Disabled (general)
- Disabled (specific subgroups)
  - Medically fragile
  - Technology dependent
  - Brain injury
  - HIV/AIDS
- Intellectual disability/developmental disability
  - Autism
  - Intellectual disability (ID)
  - Developmental disability (DD)
- Mental illness
  - Serious emotional disturbance

Target group was described descriptively and transformed into (yes/no) for each target group descriptor

**Age coverage (Appendix B-1: Specification of the Waiver Target Group[s])**

- Minimum age
- Maximum age (with either actual age or “not applicable” as age maximum if there was no age maximum presented)

The variable was then transformed into a percent pediatric coverage variable representing the percent of the age coverage that ensures those aged 0 through 21 years are covered. For example, if an autism waiver only covers children aged 1 through 6 years, then 5/21 or 23.8% of pediatric ages are covered.

**Transition (Appendix B-1: Specification of the Waiver Target Group[s])**

Due to the heterogeneity in how transition plans were described (they are a required element in the application and many were vague without specifying a specific adult waiver the child could transition to), *transition* was only given a point in the overall score if the child could age into the same waiver as an adult (ie, where there was no maximum age or the maximum age was 64 years)

**Cost containment strategies**

- Individual cost limit (yes/no): Appendix B-2: Individual cost limit. Variable transformed into either “no” cost limit or “yes” cost limit (which includes cost limit in excess of institutional costs, institutional cost limit, lower than institutional cost, or cost limit defined by the state). No individual cost limit=1 point
- Limitation in number served (yes/no): Appendix B-3: Number of individuals served Part B - Limitation on number of participants at any time. Not used in final calculation because it appears that most states limit the number on the waiver even if they do not indicate this; determined to be an unreliable indicator
- Additional limits on amount of waiver services (yes/no): Appendix C-4: Additional limits on amount of waiver services. No additional limits on amount of waiver services (ie, “not applicable in application”)=1 point

**Raw number of home and community-based services offered (Appendix C-1: Summary of services covered; C-1/C-3 Participant services and service specifications)**

Home and community-based services were represented across child/family-centric domains as yes/no in the following domains:

- Case management/care coordination/transition
- Education
- Environment/home or vehicle modifications/transportation
- Specialized equipment/assistive or adaptive technology
- Counseling/psychological support/behavior
- Caregiver/parental support/counseling/family training
- Personal care/day habilitation
- Respite
- Therapies including physical therapy, occupational therapy, vision therapy, speech, and audiology
- Nursing: skilled nursing or private duty
- Medical treatment, dietary assistance, and dental care

Created *breadth of service categories offered,* which is the number of service domains divided by the total (n=11)

**Coverage of individuals served (includes both dollars allocated and time on waiver): Appendix B-3: Number of individuals served; Appendix J: Cost-neutrality demonstration; J-1: Composite overview and demonstration of cost-neutrality formula and J-2: Derivation of Estimates**

- Individuals served (years 1-5); calculated median individuals served, which is the median of those served in years 1 through 5
- Composite dollar coverage per person per year
- Length of stay on waiver, derived from J-2 derivation of estimates per year. An overall dollar per person per year was calculated by taking the “composite dollar coverage per person per year” multiplied by the (length of stay on waiver divided by 365 days). A mean rate was also calculated as an average of years 1 through 5.
- Increase in waiver capacity over time (yes/no). Does the waiver increase in the number of individuals served, waiver length of stay, or composite dollar coverage over the 5-year waiver length? Yes=1 point.

**Wait list (directly from state officials and crowdsourced from Kidswaivers.org)**

Due to the lack of ability to compare across states, the wait list was left out of the aggregate coverage score calculation. Wait list was obtained both directly from Medicaid state administrators and from a crowdsourced resource, Kidswaivers.org. Some wait lists were reported as the number of children; however, many were combined children/adults and were not comparable.

### Analyses

Central to the study’s analytic strategy was the development of normalization criteria used to assess the overall scope of coverage of each waiver. Following data extraction, we calculated the overall coverage score based on a summation of the individual criteria for each waiver. Specifically, the overall coverage score was calculated as (Percent pediatric covered percent/100)+Transition (1 point if children can age into the existing waiver)+Individual cost limit (1 point if there is NO cost limit)+(Raw services/median raw services)+(Breadth of service categories percent/100)+Additional limits on amount of waiver services (1 point if there are NO additional limits)+Increase in waiver capacity (1 point if there IS an increase in waiver capacity over the 5-year window)+(Overall rate per person per year/median rate per person per year). Individual waiver scores were then summed and aggregated to the level of the state in order to quantify variations in scope of coverage by state and across states. States were ranked from highest to lowest coverage.

## Results

This project was funded in 2017, and data extraction was conducted in two phases: (1) waivers that were considered nonexpired through December 31, 2017, and (2) the final sample that included nonexpired waivers through October 31, 2018. Overall, 142 eligible waivers across 45 states were included in the final analysis, which is still ongoing. Five states chose other funding mechanisms and did not use the 1915(c) waivers for children. By following the process outlined for data extraction, there were no missing data for any of the waiver elements included in this analysis. Study analysis was completed in January 2019, and after analyzing each individual waiver, the results were aggregated at the level of the state and for each diagnostic subgroup. The published results are forthcoming.

While defining the HCBS scope of services for children, we established that existing criteria for HCBS taxonomy developed by Peebles and Bohl (developed for all HCBS, not specific to pediatrics) [32] should be reconsidered for waivers targeted toward children and families. Some HCBS services do not have the same level of applicability for children (eg, adult day services) and could be eliminated for waivers targeting children, while other categories could be further expanded more fully to explicate services that are most pertinent (ie, expanding education into a category and expanding caregiver support so that respite can be a category). All recommendations for child- and family-centric HCBS taxonomy are found in Table 1; these involve recommendations for modifying the existing adult HCBS taxonomy specifically for pediatric and family-centered services.

**Table 1:** Recommendations for changing existing adult home-and community-based service taxonomy to accommodate children’s waivers.

Existing adult HCBS^a^ taxonomy as defined by Peebles and Bohl [32]	Recommendations for child/family-centric taxonomy orientation
Case management	In addition to case management, consider adding Care coordination and Transition coordination
Around-the-clock services such as group living (residential habilitation and mental health), shared living, in-home residential habilitation	Group living and shared living are not readily applicable to children’s waivers because the vast majority of children reside in the home setting. However, there are some situations where these elements would be applicable.
Supported development such as job development and ongoing supportive development	These elements can remain, and supported development and can be targeted toward adolescents and young adult on child waivers.
Day services such as day habilitation, education services, day treatment, adult day health, medical day care, and community integration	These elements can remain but are not frequently encountered due to the majority targeting adult day health, etc. For child-based waivers, consider splitting out “education” as a stand-alone waiver element and one that has the ability to be synergistic with the 1115 waivers.
Nursing such as private duty nursing and skilled nursing	These elements can remain and are readily applicable.
Rent and food expenses for live-in caregiver	These were dropped from the HCBS taxonomy due to low percent reporting [32].
Home-based services such as home health aide, companion, personal care, and homemaker	Consider combining these with “day services” for child waivers.
Caregiver support such as respite and caregiver counseling/training	Consider breaking out these categories for further clarification due to the importance of the caregiver for children and families. Consider: (1) caregiver/parental support, counseling, and family training and (2) respite.
Mental health and behavioral health such as mental health assessment, crisis intervention, behavior support, and psychosocial rehabilitation	These elements can remain and are readily applicable.
Other health and therapeutic service such as prescription drugs, dental services, occupational therapy, physical therapy, respiratory therapy, cognitive rehabilitative therapy, speech, hearing, and language	Due to the nature of target groups, waivers that include children should consider breaking these out into two categories: (1) Therapies including physical therapy, occupational therapy, vision therapy, speech, and audiology and (2) Medical treatment, dietary assistance, and dental care.
Services supporting participant direction and participant training such as financial management services and information and assistance in support of participant direction	Not readily applicable to children’s waivers or families.
Equipment, technology, and modifications such as personal emergency response system, home/vehicle adaptations, and supplies	These elements can remain and are readily applicable.
Nonmedical transportation	We condensed nonmedical transportation into environment/home/vehicle modifications because the priority for the child/adolescent would be vehicle modification.
Community transition services	Not readily applicable to children’s waivers or families.

^a^HCBS: home-and community-based service.

## Discussion

We present a novel analytic methodology to systematically evaluate 1915(c) Medicaid waivers targeting CMC that can be replicated and updated as new waivers are approved. Even though there was uniformity in the Medicaid applications, there was high heterogeneity in how waiver eligibility, transition plans, and wait lists were defined. To accommodate this heterogeneity, normalization criteria for cross-waiver comparison were developed based on the ability to conduct analysis without threats of missing data, which required these important elements to be excluded in the overall coverage score. Greater data harmonization across states can allow expansion of the overall coverage score over time if these elements can be captured in systematic and reproducible ways. Additionally, another major methodological finding was the inability to capture CMC alone by focusing on the “disabled” target groups. This unanticipated challenge resulted in a broadened approach by including all waivers targeting children. The overall result of this decision will lead to a much more robust data set and likely to greater policy implications and translation.

**Figure 1:**
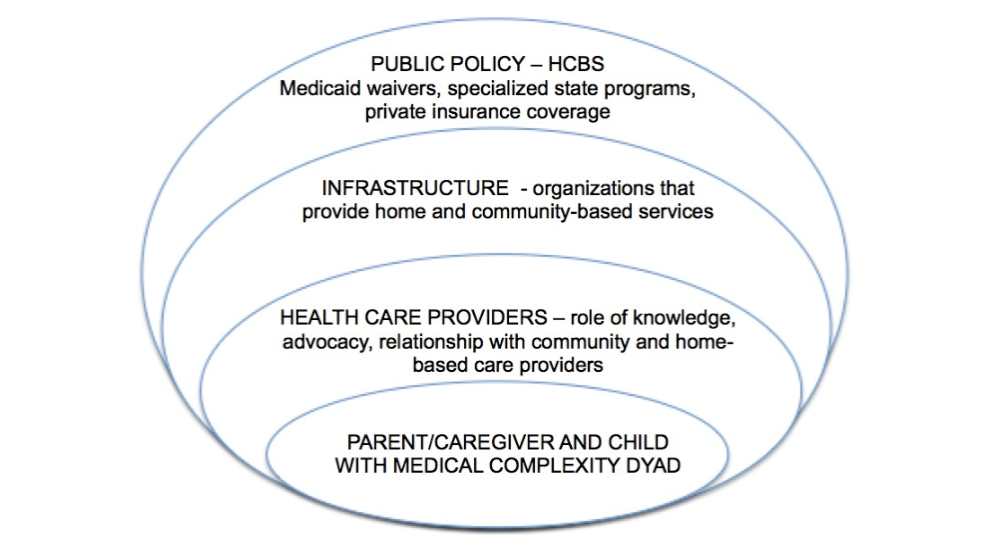
Socioecological model outlining research needs of care of children with medical complexity transitioning from hospital to home. HCBS: home and community-based services.

The 1915(c) Medicaid waivers are not the only mechanism available to fund home and community-based services for CMC, but they are, by far, the most widely used [14,33]. Even if states use a combination of 1915(c) and 1115 demonstration waivers (experimental or pilot programs that promote the objectives of Medicaid), moving to Medicaid managed care or other funding pathways, the overall coverage score can still be used as part of a composite score representing access to HCBS [18,20,34]. Although the overall coverage score represents an important first step in understanding access and differences in state interpretations, several research gaps exist. Using the socioecological model outlined in Figure 1, we believe there is a need for better links between public policy, infrastructure, health care providers, and a family-centered approach to extend this research by assessing quality outcomes related to HCBS; understanding of family-centered needs regarding timing, frequency, service extensions, preferences with respect to medical homes [35,36], and transition; and formal economic and policy evaluations of components of waiver services to understand their efficacy as well as studies related to the impact of such waivers on family functioning and economic sustainability (ie, return on investment).

## Appendix

Multimedia Appendix 1 Peer-reviewer report from the Lucile Packard Foundation for Children's Health.

## Abbreviations

CMC: children with medical complexity
HCBS: home and community-based services
