# Supplementary material for: Publicly Funded Home and Community-Based Care for Children With Medical Complexity: Protocol for the Analysis of Medicaid Waiver Applications
Source: JMIR Res Protoc. 2019 Jul 25;8(7):e13062. doi: 10.2196/13062 (PMC6686641; doi:10.2196/13062)
Supplement: Multimedia Appendix 1 [file resprot_v8i7e13062_app1.pdf]

UNIVERSITY of VIRGINIA

Jessica Malpass <jlk2t@virginia.edu>

Review of proposal to the Lucile Packard Foundation for Children's Health

Jessica Keim-Malpass <jlk2t@virginia.edu> Fri, Dec 7, 2018 at 3:09 PM
To: Jessica Keim <jlk2t@virginia.edu>

---------- Forwarded message ---------
From: **Schor, Edward** <Edward.Schor@lpfch.org>
Date: Mon, May 1, 2017 at 2:31 PM
Subject: Review of proposal to the Lucile Packard Foundation for Children's Health
To: jlk2t@virginia.edu <jlk2t@virginia.edu>, lmc8c@virginia.edu <lmc8c@virginia.edu>

Jessica and Lisa,

Our review committee met last week and had a few questions and comments about your proposal:

- What potential use could be made of the results of this project? How might it be used? Who would use it?
- The committee thought the project would provide a good baseline as we anticipate changes in state waivers.
- To what extent does the attached report obviate the need for the work you propose or potentially contribute to it?
- The amount of detailed review of materials the project entails seems extensive. Are you adequately staffed to complete this work and is your timeline realistic?
- Would it be prudent to pilot your methodology on a small sample of waivers prior to taking on the full array?
- Waivers sometimes aren't actually fully implemented, and thus described services aren't actually made available as proposed. How will your methodology consider this? Wouldn't a valid assessment of these waivers require interviewing families in each state to know what is actually happening and helping?
- You mention that the data from this project will eventually be used in formal economic evaluations. Can you say more about this?
- You also say that this study will facilitate a formal policy evaluation. We were under the impression that this project included policy evaluation.
- It may be more difficult to contact state waiver administrators than you anticipate.
- The proposal made a point of mentioning family-centered care, but it is not clear how that is connected to the proposed project or how that orientation might improve services. This aim of the project is interesting but may not be essential. Similarly, the issue of transition from pediatric to adult care is an issue of increasing interest, but might be adding work to your ambitious proposal.
- How will you assure that the algorithm you will develop to evaluate the quality of coverage of HCBS Medicaid waiver services for CMC is valid? Will you use outside expertise to guide this?
- How else might you draw conclusions from the review of waivers?
- Aren't waivers generally tied to fee-for-service payments, yet increasingly states are turning to managed care/health plans to take responsibility for long term care services? How will this analysis provide useful information in the face of this trend?
- It would be helpful if you could be more specific about the anticipated projects of this projects. For example, if you anticipate producing three papers, can you suggest the topics/titles of those papers and the journals to which they might

be submitted? If you will prepare an issue brief, who will its intended audience be and how will it be distributed.

- One key audience for this work will be state Medicaid staff, nationally. What will you want them to know and how will you reach them? Will they play any role in guiding the project?

- Data from the National Survey of Children's Health will be released to the public in late summer. Have you reviewed the content of that survey? How might it inform your work? We suspect it won't be as useful as you might hope.

We would appreciate your response to these questions and comments by May 15th. Your response may take the form of either an addendum to the proposal you have submitted or a revised proposal, whichever you prefer. Feel free to contact me if you have questions.

Ed Schor

Edward Schor, MD
Senior Vice President
Lucile Packard Foundation for Children's Health
400 Hamilton Avenue, Suite 340 | Palo Alto, CA 94301
(650) 736-2663 | Fax (650) 498-2619 | Edward.Schor@LPFCH.ORG
www.lpfch.org
www.kidsdata.org

--

Jessica Keim Malpass, PhD, RN
Assistant Professor
University of Virginia School of Nursing

Confidentiality Statement: This email message, including any attachments, is for sole use of the intended recipient and may contain confidential and privileged information. Any unauthorized review, use, disclosure, or distribution is prohibited. If you are not the intended recipient, please contact the sender by reply email and delete the original and all copies of this email.

MMRR2014_004_03_b01.pdf
522K

University of Virginia School of Nursing

May 4, 2017

Dear Dr. Schor,

Thank you for the opportunity to respond to comments from the Lucile Packard Foundation for Children's Health Review Committee. We are extremely passionate about this topic and project and appreciate the chance to be able to elucidate some concepts from the proposal. Given the recent political developments over the past months with proposed cuts to Medicaid provisions, political talk of transitioning Medicaid services to fixed block grants, and concerns over state Medicaid solvency, we are extremely committed to this research and sustained advocacy for children with medical complexity and their families.

Attached, you will find the comments from the reviewers and our responses. We have two appendices with a new timeline, budget and budget justification (based on reviewer feedback). Additionally, we have attached a biosketch for an added investigator, Dr. Christine Kennedy, Professor, School of Nursing and Medicine, University of Virginia, who is added to this project on an in-kind basis given that we already have a weekly lab meeting infrastructure. We will also attach the original proposal for ease of review.

Sincerely,

Jessica Keim-Malpass, PhD, RN

Assistant Professor

University of Virginia School of Nursing

Jlk2t@virginia.edu

The reviewer responses are the comments noted in bullets with responses labeled below.

- What potential use could be made of the results of this project? How might it be used? Who would use it?

R1: We view the impact of this study on four levels:

(1) *Comprehensive policy evaluation to establish the foundation for the current state of HCBS Medicaid waivers for CMC.* The core of this proposal is to provide a critical policy evaluation that is focused on the current scope of services of Medicaid waivers targeted towards children with medical complexity (CMC). There is currently no systematic evaluation of state Medicaid waivers for CMC and virtually no data on how various states interpret coverage and service provisions for CMC. Our research aims directly underpin the policy evaluation by (1) Describing elements of Medicaid home and community based service (HCBS) waivers targeted toward CMC; (2) Assess how states enact cost-control measures for HCBS for CMC; (3) Provide preliminary evidence on how existing models of care for HCBS can be implemented to provide family-centered care. We find this preliminary work critical in the light of profound policy changes directed at Medicaid and some of the states transitioning to Medicaid managed care and including HCBS in the capitation models.

(2) *Development of a data repository and access to data elements.* We feel very strongly that in the process of conducting the policy evaluation, the data elements that we abstract, code, and analyze must be freely available to the public (families of CMC, researchers, clinicians, policy makers). We will be working with the Center for Open Science (in Charlottesville, VA) to work through their *free* platform (because University of Virginia is a key collaborator/stakeholder) to provide the data in a usable context. Not only will this provide foundation for future policy and evaluation research, but we would love to have families of CMC directly benefit from this access as well.

(3) *Build advocacy engagement with key stakeholders.* Along with directly providing data to families of CMC, we will be working to engage with them during this process to determine their data access priorities and priorities that will not only support future funding, but also guide future research priorities. I have already started engagement in the Virginia Facebook group Moms in Motion (Medicaid Waivers Discussion Group) which is an example of one of many state based social media discussion groups that pose question and responses to certain Medicaid waiver questions targeted towards families of CMC. While these methods are not central to this proposal, the PI has specific background in internet based methods, health literacy, health communication, and online community-based participatory research. We did not want to include elements related to social media engagement in the current proposal due to vastly increasing the scope of study, but the PI is committed to disseminating findings from this study in all of the available CMC groups that she is connected to, as well as receiving iterative feedback from families of CMC. As we know, families of children with CMC have to do significant navigation to access and secure services and **they are the experts in this process.** We will be conducting this policy

evaluation **alongside their guiding expertise.** We also envision direct policy engagement with state Medicaid waiver administrators. This work will provide an evaluation of best practices, evaluation of scope of services, and explanation of **common data elements embedded within waiver services** that are critical to providing family-centered care.

(4) *Future funding.* This proposed research provides critical pilot funding for subsequent NIH level grants (page 7). Specifically, we are focusing on: (1) developing the normalization criteria (current proposal) to then specifically conduct a formal economic evaluation (more details explained below) and (2) assessing implementation through prolonged and engaged online community based participatory methods. Both of these future grants depend and hinge on the pilot data generated from this proposal.

- The committee thought the project would provide a good baseline as we anticipate changes in state waivers.

R2: We agree. Based on 2010 data, nearly 24 BILLION dollars is spent on HCBS[1] (with the majority focused on pediatric age care), yet no formal policy evaluation exists. There is a critical gap we must fill and this proposal directly responds to that gap.

- To what extent does the attached report obviate the need for the work you propose or potentially contribute to?

R3: Thank you so much for attaching this critical contribution to the field. We were aware of the work of Peebles and Bohl (2014) and neglected to cite it directly in our initial proposal. Peebles and Bohl filled a critical gap in first describing HCBS taxonomy and waiver expenditures based on 2010 Medicaid claims files. This seminal work provides a grounding taxonomy for classification overviews based on HCBS taxonomy categories and then services. We will use Peebles and Bohl as the overall structure, but then strive to extend the work by delineating by state and further classifying service provisions, waiver eligibility restrictions, time limits, enrollment capitation limits, wait list status, individual cost limits, fees charged, cost estimates, etc., specific to pediatric provision and CMC. We will also further classifying these provisions and regulations using common data elements so that our work can be extended to other researchers as well for multiple uses.

- The amount of detailed review of materials the project entails seems extensive. Are you adequately staffed to complete this work and is your timeline realistic?

R4: Thank you for addressing this comment. We have thoughtfully reflected and very much believe the scope is achievable and manageable, but want to give more time to direct engagement with state Medicaid administrators. Instead of contacting the state Medicaid officials only in the case of unclear waiver criteria or lack of transparency with publically available documents, we want to be proactive and contact all state Medicaid waiver administrators early in the study period to engage with them directly in the process. Additionally, by incorporating the data provided through their contacts, we can be more rigorous in our own data acquisition process. To ensure rigor and transparency throughout, we will publish our evaluation protocol and data abstraction process/template ahead of the finding dissemination. To incorporate this purposeful inclusion of

state Medicaid waiver administrators, we seek to extend the study period by 6 months from 12 months to 18 months. Additionally, the PI has increased percent effort on the project by 5 percent to accommodate the change in contacting all state Medicaid waiver administrators. Hence, we have updated the timeline and budget [attached as Appendix 1 and 2] to reflect these changes. Finally, Dr. Christine Kennedy, PhD, RN, FAAN was added as a Co-Investigator to this study to assist with elements related to study decisions, analysis, and dissemination [more information on her involvement provided later in the responses, as well as an attached biosketch]. Given these purposeful decisions, we feel confident that the budget and time of the proposed study directly supports the scope.

- Would it be prudent to pilot your methodology on a small sample of state waivers prior to taking on the full array.

R5: We have had experience analyzing respite waiver policy and policy regulations specific to Virginia. During that process, we have had first-hand experience with the potential challenges that exist for families navigating through the waiver process and challenges that exist for researchers making sense of how states interpret service provision. We also have pronounced clinical experience as well in terms of interfacing with families who experience barriers in accessing these services. Our own pilot engagement with the Virginia data led to conceptualize a nation-wide policy evaluation presented in this proposal. We estimate that 38 states will have 70 waivers, and believe the analysis offers a more meaningful project to analyze the full complement available.

- Waivers sometimes aren't fully implemented, and thus described services aren't actually made available as proposed. How will your methodology consider this? Wouldn't a valid assessment of these waivers require interviewing families in each state to know what is actually happening?

R6: Thank you for bringing up this important concept and we agree with the reviewer regarding implementation concerns. The issue of implementation (and effectiveness of policy) is critical and a central component of proposed future study. Before tackling the next step with understanding implementation and factors that impact implementation efforts, we **first** need to evaluate the policy provisions as they exist as law/regulation for a critical point of foundation. The initial step in conducting a formal policy analysis directly underpins our future research endeavors focused on implementation as outlined on pg. 11 of the original PDF proposal. As outlined on pg. 11, qualitative analysis of families' perceptions are critical to understanding barriers to accessing services and limitation in scope of service provision. Additionally, it is imperative to understand the impact of the waivers on healthcare outcomes and utilization, which we propose through future research using Medicaid claims data. We will make it very clear that this policy evaluation is based on policy as described, not necessarily as policy as enacted, however, it is prudent to conduct the *policy as described* study as the initial first step in this program of research. This review comment highlights why this initial policy evaluation is so needed.

- You mention that the data from this project will eventually be used in a formal economic evaluation. Can you say more about this?

R7: The PI is a collaborator with Dr. Doug Leslie at Penn State-Hershey Medical Center who is a health economist and has experience in policy analysis of 1915 waivers specifically for children with a diagnosis of autism spectrum disorder.[2] Dr. Leslie and colleagues' work provides the

foundation in method and approach for this current policy evaluation. While Dr. Leslie is not on this current pilot proposal, he and the PI are currently collaborators on pediatric hospice utilization research among CMC using claims data and he will be integrally involved in any subsequent economic evaluation. **In future studies**, we seek to (1) formally evaluate the impact of respite (in home and out of home) on health care utilization among CMC using claims data; (2) determine cost-effectiveness of respite services among CMC using claims data; (3) through family-level inquiry (a mixed methods study) determine the impact of respite on maintenance of caregiver employment/family-level financial toxicities. **Additionally**, once we have the critical foundation research evaluating the scope of services of HCBS waivers, we can also use this data point to evaluate outcomes on state-levels using claims data, i.e., conduct a difference-in-difference analysis among a states with inclusive services versus states with less comprehensive service provision and compare CMC outcomes that would be potentially impacted by HCBS services (i.e., number of inpatient admissions, costs of services, emergency department utilization, etc.). While we would not be able to suggest causation, this type of analysis has not been conducted and will offer necessary cost information that has numerous policy implications, particularly as some states are transitioning to Medicaid managed care, and there are overall political concerns about cuts to federal Medicaid dollars.

- You also say that the study will facilitate a formal policy evaluation. We were under the impression that this project included a policy evaluation.

R8: That was the PI's error. We are proposing a formal policy evaluation in this current study. In future studies, we want to undertake formal *economic* evaluations which have direct policy implications.

- It may be more difficult to contact state waiver administrators than you anticipate.

R9: We reflected a lot on this comment and agree with the reviewer. As stated in R4, we want to more purposefully engage with state waiver administrators for all states and we are going to embed this in our design as a modification. To compensate for this change and the challenges (multiple attempts) needed to contact administrators, we propose adding 6 months to the study design and the PI is increasing percent effort by 5%. Additionally, we are going to make transparency in waiver information a highlighted component of the analysis. We will essentially take this opportunity to analyze the transparency of information available (length of time for each state to access information, literacy level of application documents and available resources, and the scope of information that is available on state websites). This will give us insight on the navigation challenges that families of CMC face when trying to access these materials. These actions should not be viewed as punitive by state waiver administrators, we want to actively engage them in the process to provide feedback for best practices for increased transparency/access and will use language that views their insights as critical in this process. While we have always envisioned families of CMC as key stakeholders in the process, we also want to expand our lens to view state administrators as key stakeholders as well.

- The proposal made a point of mentioning family-centered care, but it is not clear how that is connected to the proposed project or how that orientation might improve services. This aim of the project is interesting but may not be essential. Similarly, the issue of transition from

pediatric to adult care is an issue of increasing interest, but might be adding work to your ambitious proposal.

R10: The orientation of this project is family-centered care because components of HCBS waivers provide the critical underlying supports that allow families to remain in their home settings/communities rather than institutionalized settings or the hospital, which is endorsed by the American Academy of Pediatrics, Council on Children with Disabilities.[3] We believe that the underlying body of work proposed will directly underpin these key concepts. Aim 3 is essential in providing guidance for best practices through exemplar state models of care and we believe this is a critical component of the proposed study. We will only be abstracting eligibility requirements of the adult waivers (not the full complement of analysis that will be geared toward the CMC waivers) to describe how adolescents can potentially transition to adult services through pathways provided in HCBS waivers. Transition of services has been dramatically understudied among CMC and we also feel this component is critical in providing best practice recommendations to state administrators and policy makers.

- How will you assure that the algorithm you will develop to evaluate the quality of coverage of HCBS Medicaid waiver services for CMC is valid? Will you use outside expertise to guide this?

R11: We will use the guiding principles of analysis based on the work of Leslie and colleagues[2] and will consult Dr. Leslie during development of the data extraction template and analysis. Definitions and categories of services will be extended from the work of Peebles and Bohl.[1] We have also added Dr. Christine Kennedy as a Co-Investigator to this proposal. She is added as in-kind percent effort because she is already engaged with both Drs. Keim-Malpass and Leztkus in a weekly research lab meeting focused on health literacy and CMC. If funded, the weekly lab meeting would be solely dedicated to this project. She is a current endowed professor at the University of Virginia but was a previous professor at UCSF, where she was heavily involved in pediatric research with public policy implications. In California, she was appointed as a Commissioner by the San Mateo County Board of Supervisors and offered guidance in distributing money from Proposition 10, which created the California Children and Families Commission. Dr. Kennedy is a senior nurse scientist whose research related to early childhood development has directly underpinned health policy efforts.

The PI has completed a post-doctoral fellowship in health policy, and was recently the PI on a Virginia Department of Health funded study (manuscripts under review) where we conducted a similar analysis comparing scope of services and comparative analysis among 5 different Child Development Center (early identification for pediatric behavioral and emotional diagnoses) across the state of Virginia. This proposed study is larger and across-states (not within-state), but the PI is comfortable with the proposed methodology.

Additionally, as described in our methodology (page 12 of original proposal), because we will have multiple abstractors and reviewers of data elements, we will perform duplicative data abstraction and deliver evidence of inter-rater reliability through the process and rely on normalization of quantitative data elements to reduce bias in analytic approaches. We have also decided that the

Graduate Research Assistant to be hired will be a graduate student recruited from the Batten School of Public Policy at the University of Virginia.

- How else might you draw conclusions from the review of waivers?

R12: An important addition is the addition of the transparency component of the analysis, where we will directly incorporate how available data is for families of CMC [described in R9].

- Aren't waivers generally tied to fee-for-service payments, yet increasingly states are turning to managed care/health plans to take responsibility for long term care services? How will this analysis provide useful information in the face of this trend?

R13: Yes, as noted in the original proposal, states are increasingly considering Medicaid managed care and families of CMC are very concerned about additional limitations in service provision. To date, there are very few guiding policy evaluations that can help states manage resources and make decisions regarding scope of services. This policy evaluation can offer guidance, best practices, and an overview of how various states interpret services, which in turn can be considered during changes in care provision/reimbursement.

- It would be helpful if you could be more specific about the anticipated projects of this projects. For example, if you anticipate producing three papers, can you suggest the topics/titles of those papers and the journals to which they might be submitted? If you will prepare an issue brief, who will its intended audience be and how will it be distributed.

R14: The deliverables are as follows:

(1) A **White Paper** that offers crude data elements (scope of services) and state-by-state comparative overview. This will accompany the published **data repository** and both will be freely accessible and published online through help of the Center for Open Science [free for UVA faculty]. We will engage with families of CMC widely predominantly through Facebook and Twitter and share these resources widely within the community.

(2) An **issue paper/policy brief** – geared toward the academic and policy audience. This policy brief will be submitted through traditional academic journals and will provide an overview of findings from Aims 1, 2, and 3 synthesized in form that is accessible to policy makers and families of CMC. We are purposefully seeking a high impact journal for this submission so that the policy brief can be disseminated to advocates, families of CMC, researchers, clinicians, and policy makers. The professional conference we will target will be policy-oriented in nature, likely the *National Academy for State Health Policy* annual conference so the findings can reach a wide ranging audience, including state Medicaid administrators. Additionally, the PI will write an opinion editorial to accompany this article for general audiences. See recent pediatric hospice example by PI: http://www.richmond.com/opinion/their-opinion/guest-columnists/jessica-keim-malpass-lisa-c-lindley-column-obamacare-repeal-hurts/article_00f290ae-ba4a-5506-ae17-8113d7d0d17f.html

Proposed title of policy brief: "Medicaid Home and Community Based Waivers for Children with Medical Complexity" Proposed journal: Health Affairs [submitted to their policy brief section, which is open access for policy briefs]

(3) Publication of the protocol prior to dissemination of study findings [new in amendment, but will ensure rigor and transparency of process]

Proposed title, "A protocol to evaluate scope of services of home and community based Medicaid waivers for children with medical complexity"

Proposed journal: JMIR Research Protocols [open-access]

(4)Publication of state comparison of HCBS (Aim 1)

Proposed title: "Medicaid 1915 home and community based waivers for children with medical complexity"

Proposed journal: Academic Pediatrics or PLOSOne [open-access]

(5)Publication of cost-control measures used for HCBS among CMC (Aim 2)

Proposed title: "Analysis of state waiver cost-control measures for children with medical complexity"

Proposed journal: Journal of Public Health Policy

(6) Publication of transition to adult services (Aim 3)

Proposed title: "Transition pathways for children with medical complexity using Medicaid waivers"

Proposed journal: Intellectual and Developmental Disabilities

- One key audience for this work will be state Medicaid staff, nationally. What will you want them to know and how will you reach them? Will they play any role in guiding the project?

R15: As noted in previous responses, we are offering an amendment to the design to formally include administrator engagement in the evaluation process. As noted, we believe this will strengthen our rigor and involve administrators more centrally as key stakeholders. The IRB submission will include interviewing them (with verbal consent obtained) about scope of services and follow-up to any data elements not available through publically accessible documents. The PI has extensive background in qualitative methodology, interviewing, engagement with key stakeholders and qualitative analysis [see biosketch], and this interview will be directed/ focused on scope of services for the state. By involving them directly in the process, we will offer those administrators who are willing to remain involved to be on an **advisory board** where we can review the findings of this project and the administrators can inform subsequent study development through a participatory frame. The expansion of the study by 6 months will more purposefully embed them in the process.

- Data from the National Survey of Children's Health will be released to the public in late summer. Have you reviewed the content of that survey? How might it inform your work? We suspect it won't be as useful as you might hope.

R16: We have reviewed the data dictionary of previous versions extensively and have one manuscript currently under review where we conducted a systematic review of data elements (in order to provide input for how researchers can best guide priorities for future iterations of the survey). While we agree that many of the data elements are cursory, it does offer state level data (with permission) and has access to questions related to respite and missed days from work – both of which can be outcome variables of interest. Once the policy evaluation outlined here is completed, states with high levels of scope of services can be compared to lower performers with self-reported outcome measures related to respite and caregiver missed work. Importantly, these are self-report data which offers a complementary (and needed) view that is synergistic with analyses related to claims data. Specifically, questions CfQ06_1a through 1c all are focused on respite care (in the previous 2009-2010 data release).

Appendix 1 – Updated timeline

Timeline	*Analytic & dissemination components*
Months 1-6	Hire Graduate research assistant<br>IRB exempt status obtained (non-human subjects research)<br><br>Publish evaluation protocol to ensure rigor and transparency<br><br>Retrieve all current waiver, eligibility, and cost documents. Abstract data components. Consult state legislative regulations underpinning HCBS provision within Medicaid.
Months 2-8	Make contact with all state Medicaid waiver administrators for missing or unclear data elements.
Months 8-10	Analyze normalization criteria, develop algorithm.
Months 11-12	Develop White Paper (Report) and state-by-state overview. Finalize analysis Aims 1-3. Conduct preliminary analysis on transition to adult services and potential for incorporation into medical home model.<br><br>Contact administrators who are on the **advisory board** regarding key study findings.
Months 13-18	Prepare dissemination materials (journal articles x 3, content for families for CMC, prepare for advocacy initiatives, publish White Paper (Report), publish data elements in data repository, Publish Issue Brief).

Appendix 3

References:

1. Peebles V, Bohl A. The HCBS Taxonomy: A new language for classifying home- and Community-Based services. *Medicare Medicaid Res Rev*. 2014;4(3):1-12. doi:10.5600/mmrr.004.03.b01.

2. Velott DL, Agbese E, Mandell D, et al. Medicaid 1915(c) Home- and Community-Based Services waivers for children with autism spectrum disorder. *Autism*. 2015;1915(c). doi:10.1177/1362361315590806.

3. Friedman SL, Kalichman M a. Out-of-Home Placement for Children and Adolescents With Disabilities. *Pediatrics*. 2014;134(4):836-846. doi:10.1542/peds.2014-2279.

Appendix 4 – Inclusion of Christine Kennedy, PhD, RN, FAAN Biosketch

UNIVERSITY of VIRGINIA

**Jessica Malpass <jlk2t@virginia.edu>

Proposal to the Lucile Packard Foundation for Children's Health

Jessica Keim-Malpass <jlk2t@virginia.edu>
To: Jessica Keim <jlk2t@virginia.edu>

Fri, Dec 7, 2018 at 3:09 PM

---------- Forwarded message ---------
From: **Schor, Edward** <Edward.Schor@lpfch.org>
Date: Mon, May 15, 2017 at 6:33 PM
Subject: Proposal to the Lucile Packard Foundation for Children's Health
To: Jlk2t@virginia.edu <Jlk2t@virginia.edu>

Jess,

Thank you for your revision. We have a few more questions and comments.

1. The idea of providing data to families seems unrealistic, since families are unlikely to have the capacity to do data analysis. Providing them with information based on your data analyses seems more reasonable.

2. Please describe what you mean by directly engaging with Medicaid waiver administrators? Also, do you expect that each state has such a position?

3. You propose to publish your evaluation protocol and data abstraction process/template. Is that a reasonable expectation? Do journals accept such manuscripts absent results?

4. We don't feel you addressed the inclusion of work on transition, how it relates to the larger body of work and how it will be accomplished.

5. We previously asked what you will want Medicaid staff, at the state and national level, to know from your study and how you will reach them. Creating an advisory board is a good idea, though you might want to go beyond waiver administrators and include others within Medicaid, family members, and perhaps other researchers with knowledge about this topic, e.g., Peebles and Bohl. Advisory groups are most useful when they include other than those whom you expect will agree with you on all issues.

6. You perhaps did not understand our comment about the National Survey of Children's Health. As of this year, the two national surveys, NSCH and NS-CSHNC have been combined. In the process, a number of items that previously appeared in the separate surveys may no longer be included. These exclusions may limit your ability to do the analyses you propose. You need to ascertain if that is going to be the case and explain how that might affect your plans.

Please send me your responses to these questions/comments by the end of this week.

Many thanks.

Ed

Edward Schor, MD
Senior Vice President
Lucile Packard Foundation for Children's Health
400 Hamilton Avenue, Suite 340 | Palo Alto, CA 94301
(650) 736-2663 | Fax (650) 498-2619 | Edward.Schor@LPFCH.ORG
www.lpfch.org
www.kidsdata.org

--
Jessica Keim Malpass, PhD, RN
Assistant Professor
University of Virginia School of Nursing

Confidentiality Statement: This email message, including any attachments, is for sole use of the intended recipient and may contain confidential and privileged information. Any unauthorized review, use, disclosure, or distribution is prohibited. If you are not the intended recipient, please contact the sender by reply email and delete the original and all copies of this email.

UNIVERSITY of VIRGINIA
SCHOOL of NURSING

May 16, 2017

Dear Dr. Schor,

Thank you for the opportunity to respond to additional comments from the Lucile Packard Foundation for Children's Health Review Committee. We feel strongly that the reviewer comments have strengthened our application. Attached, you will find the comments from the reviewers and our responses. We will also include the appended proposal (revision 1) for ease of review.

Sincerely,

Jessica Keim-Malpass, PhD, RN

Assistant Professor

University of Virginia School of Nursing

Jlk2t@virginia.edu

The reviewer responses are the comments noted with responses labeled below.

1. The idea of providing data to families seems unrealistic, since families are unlikely to have the capacity to do data analysis. Providing them with information based on your data analyses seems more reasonable.

Thank you for allowing us to clarify this statement. In the amendment we describe development of a data repository and access to data elements and we should have further clarified that this also entails elements of waiver characteristics that might be helpful for families to access in a centralized location (i.e. specific provisions such as, Virginia limits respite care to 140 hours per calendar year; the wait list is currently 2 years, inclusion of eligibility requirements, etc.). We would not expect them to assess the raw data unless it was in a usable form for families of varying levels of health literacy.

2. Please describe what you mean by directly engaging with Medicaid waiver administrators? Also, do you expect that each state has such a position?

Each state that uses 1915 waivers (n = 38 states) will have an administrator who oversees the waiver provisions. For example, within Virginia there is a program manager who operates within the Department of Medical Assistance Services who manages numerous CMC waivers that the PI has worked closely with in the past. Within California, Medi-Cal waiver programs are managed within California Department of Health Care Services with specific contacts and program managers that oversee waiver eligibility and implementation. Each state will operationalize the position slightly differently, but these positions are usually within the state health departments and are found using state websites. We will include contact with the administrators (and verbal consent procedures) as a part of our IRB application so we can directly initiate contact and interview them for study purposes through telephone interviews. We will ask directed questions pertaining to updated waiver wait list status, any recent changes to waivers, and current state implementation strategies. The PI has extensive background in qualitative methodologies, interviewing, and working with state health departments. Interviews will be audio recorded and transcribed verbatim. The questions posed to the administrators will be analyzed through directed content analysis achieved through line by line coding using our a priori analytic framework.[1] At the time of conversation, we will also ask the state administrators if they are interested in participation in our advisory board and subsequent follow-up.

3. You propose to publish your evaluation protocol and data abstraction process/template. Is that a reasonable expectation? Do journals accept such manuscripts absent results?

We find this expectation to be very reasonable particularly in light of the recent shift to enhanced rigor and transparency in the research and evaluation processes.[2] We outlined a potential journal for publication [*JMIR Research protocols*, journal states they publish peer-reviewed openly accessible research ideas and grant proposals, study and trial protocols, current methods and approaches, etc.] where the journal specifically focuses on publication of protocols prior to finding/results dissemination. This approach has garnered support from investigators publishing systematic review protocols, clinical trial protocols, comparative effectiveness protocols and evaluation protocols to ensure that a priori assumptions and methodologies are accounted for prior to dissemination of the results. Beyond JMIR Research protocols, there are other journals that have focused on these types

of publications including the journal *Trials* [impact factor 1.859, journal states that they publish articles on general trial methodology as well as protocols], and other similarly scoped journals.

4. We don't feel you addressed the inclusion of work on transition, how it relates to the larger body of work and how it will be accomplished.

Based on the framework of Dr. Shor (2015)[3], transition has been largely conceptualized within the medical community as collaboration and co-management between care providers within the levels of primary and specialty care. Very little attention has been focused on transition in the context of home and community based services.

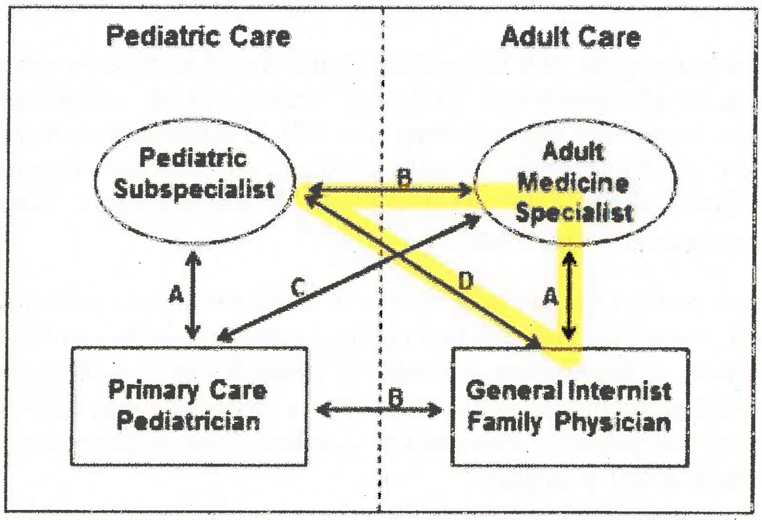

FIGURE 1
Structural basis for collaboration and comanagement in care transition. The highlighted triangle outlines the comanagement that, although unusual and unstudied, could provide lifelong care for youth with chronic pediatric illness after transition from pediatric primary care.

[Attribution for transition figure: Shor, 2015[3], citation in references]

Because so many of the pediatric-focused waivers have 18 or 21 as upper age limits, we worry about large gaps in service provision for home and community-based services once youth age-out of the waiver age eligibility. The way that the transition element would be operationalized is by assessing feasible care pathways for children to transition while enrolled on waiver-based community based services. For example, 10 states have 1915 waivers specifically for diagnoses of autism spectrum disorder that are limited to ages less than 18 or 21.[4] For those states with an ASD waiver, we would want to assess what potential adult waivers exist that youth could transition to (an adult waiver often requires re-application if such a waiver even exists). We would follow along the same analysis for any relevant pediatric waiver included in Aims 1 and 2 to determine the upper age limit of eligibility. We would incorporate an assessment of any relevant adult waivers that youth/adolescents could transition to or if there is an abrupt lack of services once the youth age out of the age eligibility. We would not assess the full complement of data elements for adult waivers, but rather offer vital (and novel) information about whether youth could transition to adult waivers

and potential limitations in access. Thus, we would be extending the transition frameworks to also consider transition elements embedded in home and community based services. The PI has experience assessing how some states use 1915 waivers to extend hospice eligibility and concurrent curative care[5] and we have noticed dramatic differences in access availability among youth/young adults who are older than 21. While not a central component of the policy evaluation, the transition piece is critical to understand the general needs and gaps in services for families of CMC with youth who will be aging out of pediatric service provision.

5. We previously asked what you will want Medicaid staff, at the state and national level, to know from your study and how you will reach them. Creating an advisory board is a good idea, though you might want to go beyond waiver administrators and include others within Medicaid, family members, and perhaps other researchers with knowledge about this topic, e.g., Peebles and Bohl. Advisory groups are most useful when they include other than those whom you expect will agree with you on all issues.

We whole-heartedly agree with this statement and would want to include a diverse range of key stakeholders in the advisory board, including; families of CMC, clinicians, representatives from key community based care representation (i.e. ARCH National Respite Network, National Hospice and Palliative Care Organization), health service researchers with knowledge about this topic (Peebles and Bohl, Leslie[6], etc.), and state Medicaid administrators. We also believe that the diversity in representation is critical.

6. You perhaps did not understand our comment about the National Survey of Children's Health. As of this year, the two national surveys, NSCH and NS-CSHNC have been combined. In the process, a number of items that previously appeared in the separate surveys may no longer be included. These exclusions may limit your ability to do the analyses you propose. You need to ascertain if that is going to be the case and explain how that might affect your plans.

Thank you for clarifying the original comment. We do have a call into the Director of the upcoming combined NSCH and the NSCSHCN and have left a message for clarification. The variables of interest pertaining to respite are core MCHB Indicators, so we would hope that they would be included in the combined version. That said, we feel as though the review committee is correct in that we need to assume they are not going to be available.

While we outlined several grant approaches in both the original application and amendment, we do feel strongly that a grant relying on self-report data from families of CMC is critical. If the NSCSHCN has eliminated questions that would meet these needs, we would work with the UVA Center for Survey Research in a subsequent grant application to develop a two-state comparative survey approach, likely Virginia and California. Virginia and California have very different levels of waiver services available for families (California has been a leader in many of these policy initiatives). We would work within provider networks to survey families of CMC and elicit the responses of interest directly. While this approach would not include all representative states, we could elicit more detailed feedback from families and still have a comparative/policy approach.

References:

1. Hsieh H-F, Shannon SE. Three approaches to qualitative content analysis. *Qual Health Res*. 2005;15(9):1277-1288. doi:10.1177/1049732305276687.
2. Buck S. *Transparency for Clinical Trials and NIH-Sponsored Research.*; 2015. http://www.arnoldfoundation.org/wp-content/uploads/2015/05/Transparency-for-Clinical-Trials-and-NIH-Sponsored-Research1.pdf. Accessed: May 16, 2017.
3. Schor EL. Transition: Changing Old Habits. *Pediatrics*. 2015;135(6):958-960. doi:10.1542/peds.2014-3934.
4. Merryman MB, Miller NA, Shockley E, Eskow KG, Chasson GS. State adoption of Medicaid 1915(c) waivers for children and youth with Autism Spectrum Disorder. *Disabil Health J.* 2015;8(3):443-451. doi:10.1016/j.dhjo.2015.03.003.
5. Keim-Malpass J, Hart TG, Miller JR. Coverage of palliative and hospice care for pediatric patients with a life-limiting illness: a policy brief. *J Pediatr Heal Care*. 2013;27(6):511-516. doi:10.1016/j.pedhc.2013.07.011.
6. Velott DL, Agbese E, Mandell D, et al. Medicaid 1915(c) Home- and Community-Based Services waivers for children with autism spectrum disorder. *Autism*. 2015;1915(c). doi:10.1177/1362361315590806.
